# Relative effects of urbanisation, deforestation, and agricultural development on mosquito communities

**DOI:** 10.1007/s10980-023-01634-w

**Published:** 2023-03-20

**Authors:** Antoine Perrin, Francis Schaffner, Philippe Christe, Olivier Glaizot

**Affiliations:** 1 Department of Ecology and Evolution, University of Lausanne, UNIL-Sorge, Biophore, 1015 Lausanne, Switzerland; 2 National Centre for Vector Entomology, Institute of Parasitology, University of Zürich, 8057 Zurich, Switzerland; 3 Francis Schaffner Consultancy, 4125 Riehen, Switzerland; 4 Museum of Zoology, 1014 Lausanne, Switzerland

**Keywords:** Landscape anthropisation, Scale of effect, Mosquito abundance, Species diversity, Culicidae

## Abstract

**Context:**

Despite numerous studies that showed negative effects of landscape anthropisation on species abundance and diversity, the relative effects of urbanisation, deforestation, and agricultural development as well as the spatial extent at which they act are much less studied. This is particularly the case for mosquitoes, which are the most important arthropods affecting human health.

**Objectives:**

We determined the scale of effect of these three landscape anthropisation components on mosquito abundance and diversity. We then assessed which landscape variables had the most effect as well as their independent positive or negative effects.

**Methods:**

We used mosquito data collected by Schaffner and Mathis (2013) in 16 sampling sites in Switzerland. We measured forest, urban and agricultural amounts in 485 concentric landscapes (from 150 to 5000 m radius) around each sampling site. We then identified the spatial extent at which each landscape metric best predicted abundance and diversity of mosquito species and compared the effect size of each landscape component on each response variable.

**Results:**

In Switzerland, urbanisation and deforestation have a greater influence on mosquito diversity than agricultural development, and do not act at the same scale. Conversely, the scale of effect on mosquito abundance is relatively similar across the different landscape anthropisation components or across mosquito species, except for *Culex pipiens*. However, the effect size of each landscape component varies according to mosquito species.

**Conclusion:**

The scale of management must be selected according to the conservation concern. In addition, a multi-scale approach is recommended for effective mosquito community management.

**Supplementary Information:**

The online version contains supplementary material available at 10.1007/s10980-023-01634-w.

## Introduction

Currently, it is well recognised that landscape anthropisation negatively impacts biodiversity worldwide (Haddad et al. 2015; Newbold et al. 2015, 2016; Raven and Wagner 2021). However, the relative effects of the main landscape anthropisation components (i.e., urbanisation, deforestation, and agricultural development) remain poorly understood. This is partly due to high interaction and correlation among these different landscape components, as one can be the cause or the consequence of the others (Tilman et al. 2001; DeFries et al. 2010; Hosonuma et al. 2012; Busch and Ferretti-Gallon 2017; Curtis et al. 2018; Nathaniel and Bekun 2020; Kadoya et al. 2022). For example, agricultural development can lead to both deforestation and the construction of roads and buildings, and urban expansion can increase demand for deforestation. Although the causality can run in both directions since deforested land can serve to build urban or agricultural areas (Busch and Ferretti-Gallon 2017). Nevertheless, each of these landscape components have their own effects. Urbanisation, deforestation, and agricultural development respectively lead to a loss of natural habitat, an increase in human density which leads to a high use of resources and waste production, and an increase in chemical pollution and disruption of natural cycles (water or nutrients). It is also essential to understand which landscape component affects biodiversity the most, because the political and practical conservation measures to be applied can differ according to the targeted landscape gradient (IPBES 2019). For instance, a first conservation policy option can be focused on reducing the rent from extensive agriculture to minimise the overall agricultural area, but a second option can be focused on increasing extractive or protective forest rent to maximise the overall forest area (Angelsen 2010).

To correctly estimate the effects of landscape variables on biodiversity, it is necessary to use the appropriate spatial extent around sampling sites (i.e., the scale of effect; Jackson and Fahrig 2012). However, the scale of effect depends on both species and the landscape variable studied (Smith et al. 2011; Miguet et al. 2016; Martin 2018). In fact, the scale at which a species responds to landscape variables depends on its mobility (Miguet et al. 2016). However, even for a species-landscape variable combination, the scale of effect can differ for different response variables, it is therefore better to empirically determine the scale of effect, rather than to predict it a priori (Moraga et al. 2019).

Despite a large literature on the effects of landscape anthropisation on mosquito abundance and diversity (reviewed by Sallam et al. 2017; Burkett-Cadena and Vittor 2018; Perrin et al. 2022), there are few data concerning the relative effects of the three landscape components as well as their scale of effect. However, there are specific effects associated with each component of landscape anthropisation on mosquito communities. For instance, Norris (2004) and Vora (2008) highlighted that deforestation favoured mosquito species with higher vectorial capacities; urbanisation created many man-made breeding sites and refugia for species capable of using them, as well as a stable source of water during the dry season due to watering and presence of flooded pipes underneath the streets; and agricultural development led to ideal local environments (e.g., higher sedimentation, shallowest water depth) and climate (e.g., warmer temperature) for mosquitoes. In addition, mosquito life cycle is complex since mosquito larvae develop in aquatic habitats, often small and/or temporary water ponds or puddles, whereas the adults live in terrestrial habitats (Becker et al. 2020). The spatial extent of the environment encountered by a larva is therefore much smaller than the extent encountered by an adult. Moreover, the daily movements linked to breeding and/or foraging of adult mosquitoes is low (median distance around 400 m; based on 35 species) while the dispersal capacity is higher (median distance around 2200 m; based on 105 species; Verdonschot and Besse-Lototskaya 2014). Miguet et al. (2016) predicted a lower scale of effect of landscape variables that most strongly affect (i) foraging success rather than dispersal success and (ii) less-mobile life stage rather than more-mobile life stage. It is thus important to determine the scale of effect and the relative effects of urbanisation, deforestation, and agricultural development on mosquito species to fully understand how landscape changes affect them. This will allow effective landscape management to limit nuisances linked to these species.

In this study, we used mosquito field data, collected and described in Schaffner and Mathis (2013), characterising the spatio-temporal diversity of the mosquito fauna in Switzerland. In this country, mosquito-related nuisance is mainly caused by their roles as vectors of pathogens of veterinary importance (Steinmetz et al. 2011; Glaizot et al. 2012; Wagner et al. 2018; Kubacki et al. 2020). Although there is still no report of local transmission of dengue or chikungunya in Switzerland, in neighbouring countries (Italy and France), several outbreaks have been reported (Schaffner and Mathis 2014; Kubacki et al. 2020; Cochet et al. 2022). In addition, the rapid colonisation of *Aedes albopictus* in Switzerland and other European countries considerably increases the risk of vector-borne disease emergence in the future (Ravasi et al. 2020, 2022). Here, we determined the scale of effect of urbanisation, deforestation, and agricultural development on mosquito abundance and diversity to evaluate whether it is consistent across different landscape variables. We then compared the effect size obtained at the scale of effect for each of these three landscape components to assess which landscape variables had the most effect on mosquito abundance and diversity as well as their independent positive or negative effects.

## Material and methods

### Field sampling

**Fig. 1:**
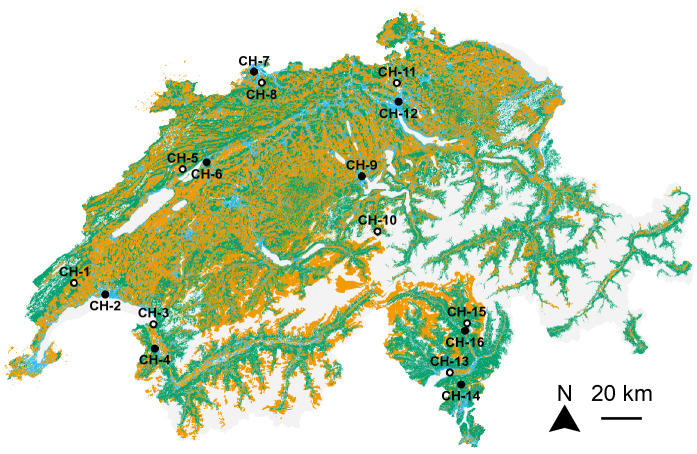
Location of the 16 sampling sites across Switzerland. Black and white dots indicate sampling sites dominated by either human-modified or natural landscapes, respectively. Orange, green, blue, and grey colours represent agricultural (i.e., *Landw. Bewirtschaftung: Nutzungsflächen* shapefile—geodienste.ch), forest (i.e., entities ‘Gebueschwald’, ‘Wald’, ‘Wald offen’, and ‘Gehoelzflaeche’ from the *Bodenbedeckung* shapefile—swissTLM3D—swisstopo), urban (i.e., *Gebaeude_Footprint* shapefile—swissTLM3D—swisstopo) and other (mainly lakes and mountains) areas, respectively. The aerial images and data provided by the swiss cantons used to generate this map were produced between 2012 and 2022. CH-1 = Mollens (VD); CH-2 = Lausanne (VD); CH-3 = Noville (VD); CH-4 = Collombey‐Muraz (VS); CH-5 = Prêles (BE); CH-6 = Biel/Bienne (BE); CH-7 = Binningen (BL); CH-8 = Arlesheim (BL); CH-9 = Luzern (LU); CH-10 = Engelberg (OW); CH-11 = Oberglatt/Winkel (ZH); CH-12 = Zürich (ZH); CH-13 = Locarno (TI); CH-14 = Camignolo (TI); CH-15 = Malvaglia (TI); and CH-16 = Locarno (TI)

Study sites, mosquito collection methods, as well as information and discussion about the completeness of data have previously been described in detail elsewhere (Schaffner and Mathis 2013) so, for brevity, are summarised here. This study was carried out in 16 sampling sites in 8 cantons across Switzerland: One sampling site in each of the cantons of Luzern (LU), Obwalden (OW), and Valais (VS); two in the cantons of Basel-Landschaft (BL), Bern (BE), and Zürich (ZH); three in the canton of Vaud (VD); and four in the canton of Ticino (TI; Fig. 1). These sampling sites were selected to include landscapes that were dominated by either natural (8 sampling sites) or human-modified (8 sampling sites) landscapes. Field sampling occurred between May and September from 2011 to 2012. Each sampling site was sampled over 5 sessions (in July 2011, September 2011, May 2012, July 2012, and September 2012). A possible effect of seasonality was tested by excluding data collected in May to focus the analyses on summer sampling (i.e., during the peak of activity of mosquitoes in Switzerland). The results were relatively similar, we therefore presented only analyses conducted on the complete dataset hereafter.

For each sampling session, potential mosquito larval habitats within the 150 m range of the focal sampling point were inspected. In positive larval habitats, larvae and pupae were collected with a dipper or a net in the water stratum according to a well-defined protocol (Schaffner and Mathis 2013). All larval specimens were stored in 70% ethanol while pupae were kept alive in a sample bottle and reared in the laboratory until emergence of adults. Complementary to immature stages trapping, two CO_2_-baited traps (CDC Miniature Light Trap or BG-Sentinel Trap (Biogents AG, Regensburg, Germany)) were placed at least one hour before sunset and recovered at least one hour after sunrise to trap adult mosquitoes during the 5 sampling sessions. Adult and 4th instar mosquito larvae were morphologically identified to the species level using methods described in Becker et al. (2010) and Schaffner et al. (2001).

### Landscape characterisation

To estimate the amount of the three habitats (forest, urban and agricultural), we calculated the percent of forest area, the percent building cover (building footprint), and the use area of agricultural holdings in the local landscape of each sampling site. Using the swissTLM3D geodata version 1.8 provided by the Federal Office of Topography swisstopo (https://www.swisstopo.admin.ch), the building footprint and the forest cover were defined with the *Gebaeude_Footprint* shapefile and the entities ‘Gebueschwald’, ‘Wald’, ‘Wald offen’, and ‘Gehoelzflaeche’ from the *Bodenbedeckung* shapefile, respectively. The agricultural cover was defined with the *Landw. Bewirtschaftung: Nutzungsflächen* shapefile version 1.4 available upon request from Swiss cantons (https://www.geodienste.ch/).

Forest, urban and agricultural amounts were measured within concentric buffers from 150 to 5000 m radius increasing the radius by 10 m each time around each sampling site (for a total of 485 buffer sizes). The smaller landscape size (150 m radius buffer) was set to include all the locations of larval habitat and adult mosquitoes sampled. The largest landscape (5000 m radius buffer) was established to both include an area higher than the median dispersal capacity of mosquitoes (Verdonschot and Besse-Lototskaya 2014) and avoid a high overlap among buffers.

### Data analysis

We considered mosquito abundance and diversity to characterise mosquito communities in the 16 sampling sites. Mosquito diversity was measured through the Shannon index, and mosquito abundance was defined for each mosquito species by the total number of individuals divided by the sampling size (i.e., the number of larval habitats and CO_2_-baited traps sampled over the 5 sessions).

We assessed the scale of effect of each landscape component on mosquito abundance and diversity independently for urbanisation, deforestation, and agricultural development gradients within the 485 buffer sizes. We used simple linear regression models and estimated the scale of effect for each landscape component—response variable combination as the buffer size where the model has the highest proportion of deviance explained (R^2^). To determine the uncertainty around the selected scale of effect, we randomly re-sampled the data with replacement (bootstrap) for each landscape component—response variable combination. Finally, we conducted multiple regression analyses to determine the sign (positive or negative) and the relative effect size of each landscape component on mosquito abundance and diversity both with the selected scale of effects and with an a priori scale of effect of 400 m (corresponding to the median daily movements of mosquitoes; Verdonschot and Besse-Lototskaya 2014).

All analyses were performed with the *vegan*, *rgdal*, *rgeos*, *raster*, and *lme4* packages available in R software (R Core Team 2022).

## Results

Overall, 11,989 mosquito specimens belonging to 15 species, 3 sibling species (*Aedes annulipes/cantans*, *Ae. cinereus/geminus*, *Culex pipiens/torrentium*) and the *Anopheles maculipennis* complex were sampled in the 16 sampling sites (mean (± SD) per sampling site = 749 ± 385 mosquitoes; Schaffner and Mathis (2013)). The most abundant species in a decreasing order of abundance were *Cx. pipiens/torrentium*, *Cx. hortensis*, *Ae. japonicus*, *An. maculipennis* complex members, *Ae. vexans*, *Ae. cinereus/geminus* and *Ae. sticticus*, accounting for a total of 97% of the sampled mosquitoes. We tested the effect of the three landscape components on mosquito species abundance only on these common species, the remaining 3% of species did not have a sufficient number of individuals per sampling site to conduct analyses with sufficient statistical power.

**Fig. 2:**
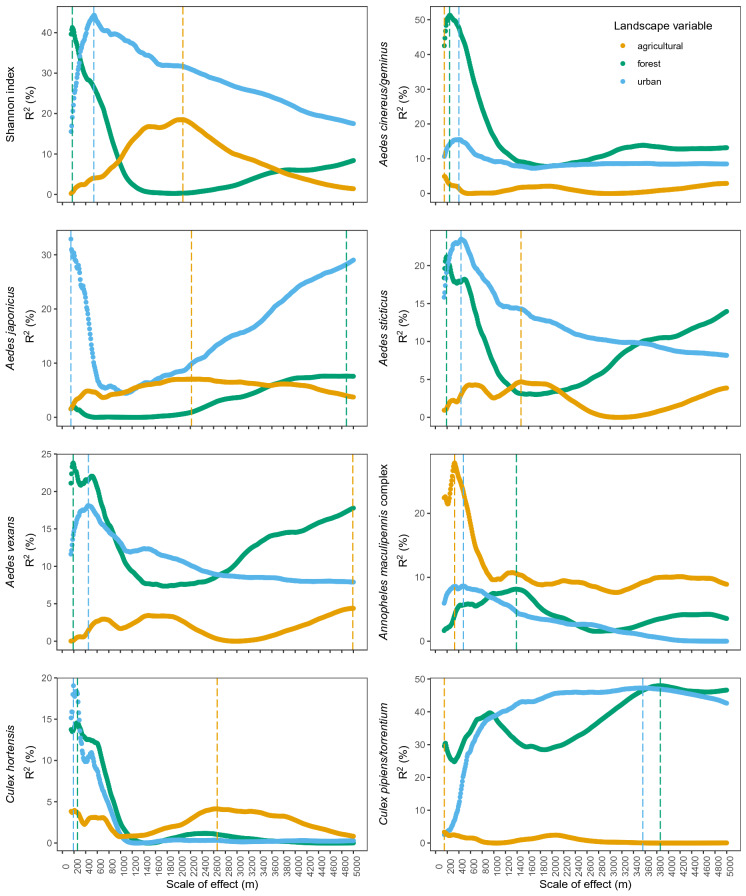
Proportion of explained deviance (R^2^) of each response variable by each landscape component according to the scale at which the effect is measured. Vertical dash bars represent the selected scale of effect (where R^2^ is the highest)

Our results show three clear peaks for the scale of effect of each landscape component on mosquito diversity. The forest amount explains a maximum of mosquito diversity variation when measured in a buffer of 200 m radius, while for the urban and agricultural amount, the explained variance of mosquito diversity is maximum when the buffer is 500 m and 2000 m radius, respectively (Fig. 2). For the scale of effect of each landscape component on mosquito species abundance, we find only one clear peak for one or two landscape components, which differ according to the mosquito species. Moreover, when the peak is clear, it suggests a scale of effect between 150 and 400 m for the three landscape components, except for *Cx. pipiens/torrentium* abundance. For this species group, the scale of effect suggests a better explained variance of its abundance by forest and urban amounts on a larger scale (Fig. 2).

The scale of effect selected for each landscape component—response variable combination varies with bootstrap resampling. However, for most of these combinations, we find a distribution of the selected scale of effect through the 1000 resampling close to a normal distribution centred on the observed scale of effect for forest and urban amounts. For the scale of effect of agricultural amount, we do not find a normal distribution centred on the observed scale of effect (except for the Shannon index) suggesting a large uncertainty in the scale of effect of agricultural development on mosquito abundance (Fig. S1).

**Fig. 3:**
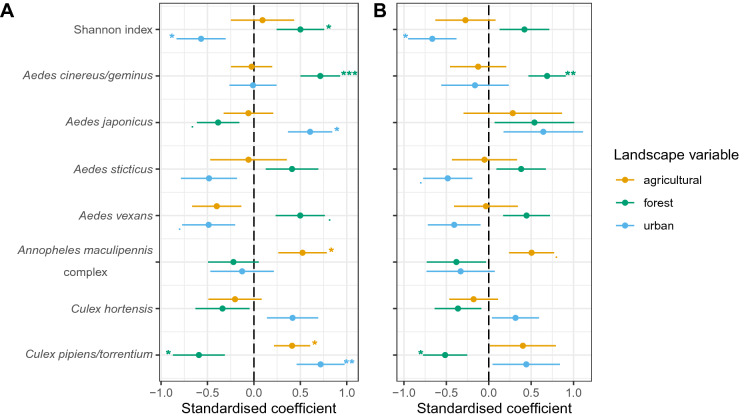
Effect size (standardised regression coefficient ± SE) of each landscape variable on mosquito diversity and species abundance. The effect size is calculated at **A** the scale which maximises the explained deviance of each response variable (R^2^) by each landscape component; **B** an a priori scale of effect of 400 m. p ≤ 10; *p ≤ .05; **p ≤ .01; ***p ≤ .001

Regarding landscape component effect sizes, we find a significant effect of similar size for forest and urban amounts on mosquito diversity, while the effect of agricultural amount is small and not significant. Our results show that mosquito diversity decreases in response to deforestation and urbanisation (Fig. 3A). The size and sign of the effect of landscape components on mosquito abundance depend on the species studied. For *Ae. cinereus/geminus*, we only find a high positive effect of forest amount, while for *Ae. japonicus* and *An. maculipennis* complex, we only find a high positive effect of urban and agricultural amounts, respectively. For *Cx. hortensis*, *Ae. vexans* and *Ae. sticticus* abundance, there is no significant effect of the three landscape components despite a trend suggesting a positive effect of urbanisation and deforestation for *Cx. hortensis* abundance and a negative effect of these two landscape components on *Ae. sticticus* and *Ae. vexans* abundance. In addition, landscape anthropisation has a positive influence on *Cx. pipiens/torrentium* abundance irrespective of the landscape component (Fig. 3A). Finally, the effect of landscape anthropisation on mosquito abundance is both positive (for four species: *Ae. japonicus*, *An. maculipennis* complex, *Cx. hortensis* and *Cx. pipiens/torrentium*) and negative (for three species: *Ae. cinereus/geminus*, *Ae. sticticus* and *Ae. vexans*). Our results also show that many landscape effects are not detected if an a priori estimate of the scale of effect is used, whether on mosquito diversity or abundance (Fig. 3B).

## Discussion

In the present study, we analysed a field dataset of mosquitoes collected from 16 sampling sites in Switzerland to determine the scale of effect of three landscape anthropisation components and to assess which of these had the most effect on mosquito abundance and diversity. We showed that landscape anthropisation leads to an overall decline of mosquito diversity in Switzerland, which is in line with most studies conducted in other parts of the world (reviewed by Perrin et al. 2022). This result is generally explained by the presence of more favourable breeding environments, such as freshwater and brackish water wetlands (Ferraguti et al. 2018), or more diverse host species (Newbold et al. 2014) for insects in forests compared to urban and agricultural areas. Our results go further and suggest a greater effect of deforestation and urbanisation on mosquito diversity compared to agricultural development. Moreover, the effect of deforestation acts on a smaller scale than urbanisation. According to Miguet et al. (2016), the effect of these two landscape variables would therefore act on different biological factors. Deforestation might lead to the elimination of natural sites for laying eggs and/or a reduction in host density and thus acts on mosquito breeding/foraging behaviour, while urbanisation might impact mosquito dispersal by acting as a barrier. Forest amount could also have a positive effect on larval development, while urban areas could negatively affect adults. However, based on our exhaustive literature search, all these hypotheses are yet to be tested because most studies only focused on one landscape gradient without considering the others. It is therefore not possible to conclude on the independent effects of deforestation and urbanisation from these data.

Concerning mosquito abundance, the clear scale of effects of landscape anthropisation are between 150 and 400 m for most species. This range of scale of effects corresponds approximately to the distance travelled by mosquitoes during their daily movements linked to breeding and/or foraging (Verdonschot and Besse-Lototskaya 2014). The effects of landscape anthropisation therefore act at a specific scale and landscape management decisions should be considered accordingly. However, the important landscape variables, and their positive or negative effects, vary depending on the species. These results are not surprising given the variety of mosquito ecological characteristics, especially the difference in larval habitat preference (Almeida et al. 2020), as well as in feeding behaviour. Some species feed more on humans, by definition more present in anthropised environments, while others feed on other animals (e.g., birds or amphibians) more present in natural environments (Becker et al. 2020). In addition, these different landscape variables of importance correspond to the known characteristics of the species studied (Becker et al. 2020). For example, *Ae. cinereus/geminus* is a forest species, although it can be found in a wide variety of habitats, and our result showed a main positive effect of forest amount on this species. Similarly, *An. maculipennis* can be found in agricultural areas (e.g., in rice fields, artificial pools or ditches; Becker et al. 2020) and are positively affected by agricultural development. There are exceptions such as the significant positive effect of urbanisation on *Ae. japonicus* which is known to be a forest species (Becker et al. 2020) but also to be able to adapt to an urbanised environment by growing in a large panel of artificial containers. The composition of mosquito communities in Switzerland is therefore very heterogeneous depending on the environment. Interestingly, we found that the three main potential disease vectors (i.e., *Ae. japonicus*, *An. maculipennis* and *Cx. pipiens*) are positively affected by landscape anthropisation. This result agrees with the growing body of studies showing an increase in mosquito species abundance which are vectors of human diseases and an opposite pattern for almost all other species in anthropised areas (reviewed by Burkett-Cadena and Vittor 2018; Perrin et al. 2022).

The response pattern of *Cx. pipiens/torrentium* abundance to landscape anthropisation is different than the others. This species is affected by the environmental conditions similarly across all scales and the three landscape components have a significant effect. In addition, urbanisation and deforestation affect positively *Cx. pipiens/torrentium* abundance at a larger scale than other species. Several factors could partly explain why this mosquito species has an abundance that is not scale sensitive. First, *Cx. pipiens* is an opportunistic feeder on avian or mammalian hosts, when compared to other more specialised species feeding exclusively on mammals, birds or amphibians (Schaffner et al. 2001; Becker et al. 2020). Second, this species is characterised by its ability to exploit a large panel of potential habitats, from natural to anthropised areas (Gad et al. 1995; Becker et al. 2020; Wilkerson et al. 2021). It is also known that dispersal distance is a good predictor and is positively correlated with the scale of effect (Jackson and Fahrig 2012). However, the dispersal capacity of *Cx. pipiens* is a controversial issue, some authors identify it as a species with the higher dispersal capacity within our set of mosquito species (Verdonschot and Besse-Lototskaya 2014) and others describe it as having a short active dispersal range (e.g., Ciota et al. 2012; Hamer et al. 2014; Becker et al. 2020). In addition, other species with a high dispersal capacity, as *Ae. sticticus*, show a different response pattern than *Cx. pipiens*. Although our data does not provide underlying causes of this pattern, our results suggest that landscape management should be multi-scale when conservation goals are focused on *Cx. pipiens*.

Our study has several implications for research. First, without an approach to determine the scale of effects, we would not have highlighted all significant effects of landscape anthropisation. This suggests that future studies should empirically estimate the scale of effect using a multi-scale design to estimate landscape effects on a biological response. Second, we found an effect of the three landscape components and studies focusing on a single one could miss a potentially significant effect of landscape anthropisation on biodiversity. Finally, we showed a significant effect of urbanisation and deforestation on mosquito diversity but not at the same scale. This result is in line with numerous empirical studies suggesting that the scale at which the landscape acts on a biological response depends on the landscape variable being measured (see Miguet et al. (2016) for references).

In conclusion, there is no simple guideline for landscape management and planning because the scale of management depends on the biological response and the species considered which must be selected according to the conservation concern. In the case of mosquitoes in Switzerland, if the management focuses on the increase of mosquito diversity to maintain high interspecific competition and thus limit the proliferation of disease vector species, it is necessary to maximise forest areas and limit highly urbanised areas in the few hundred meters around mosquito breeding areas. However, if the conservation concerns involve *Cx. pipiens*, an important vector of many viruses and other pathogens and the most abundant mosquito species in our study areas, it is also necessary to maximise forests and limit highly urbanised areas around mosquito breeding sites, but on a much larger scale.

## Supplementary Information

Below is the link to the electronic supplementary material.

Supplementary file1 (PDF 100 KB)

## Data Availability

Raw data and R code are available in Mendeley Repository (DOI: 10.17632/cd5k3xt4w5.1).
