# Supplementary material for: Relative effects of urbanisation, deforestation, and agricultural development on mosquito communities
Source: Landsc Ecol. 2023 Mar 20;38(6):1527–36. doi: 10.1007/s10980-023-01634-w (PMC10203030; doi:10.1007/s10980-023-01634-w)
Supplement: Supplementary file 1 — Supplementary file1 (PDF 100 KB) [file 10980_2023_1634_MOESM1_ESM.pdf]

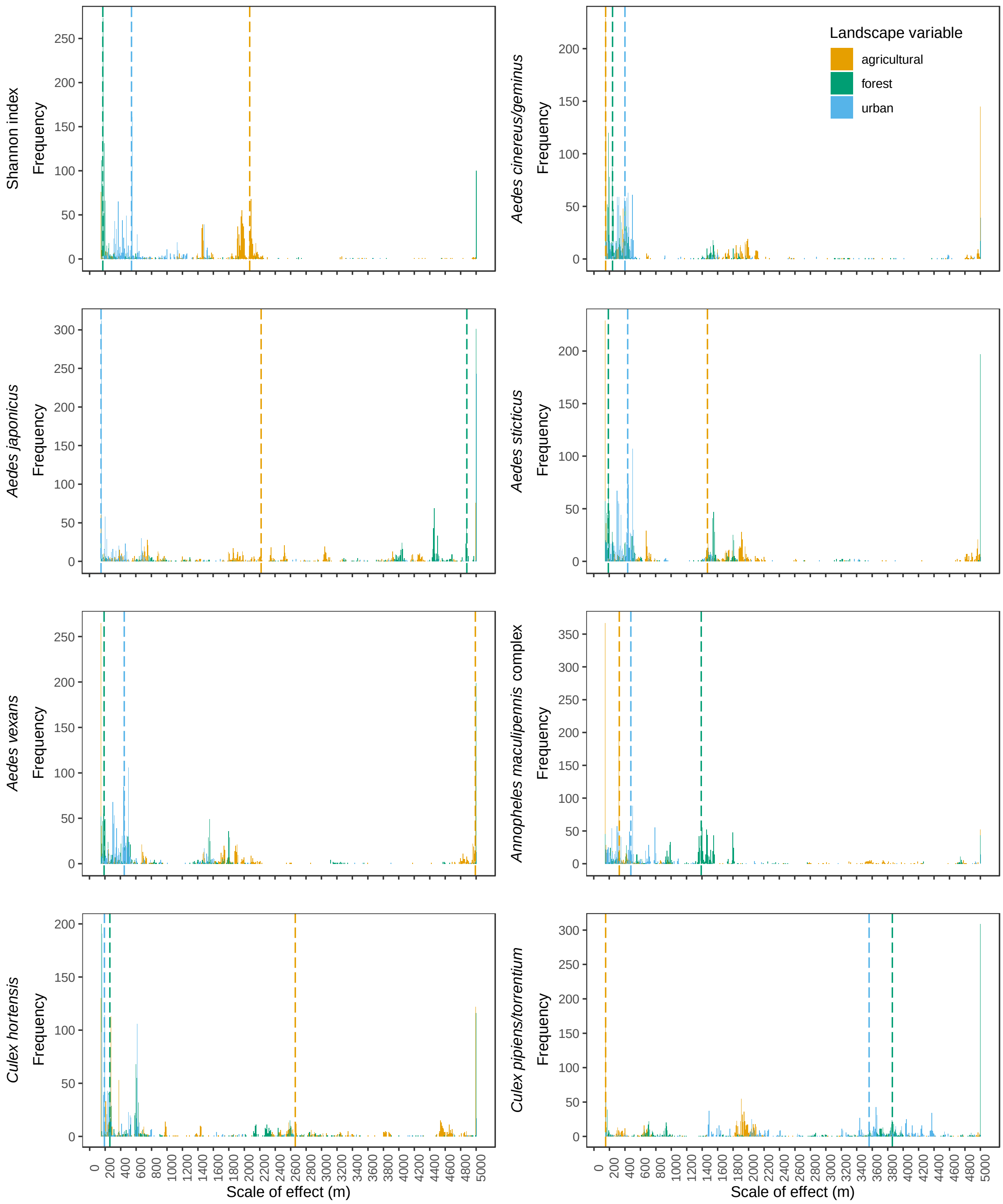

Fig. S1: Uncertainty around the selected scale of effects, estimated by bootstrapping. For each response variable - landscape component combination, we randomly re-sampled with replacement the 16 sampling sites 1000 times. We then selected the scale of effect for each resampled data set. The frequency corresponds to the total number of times that each spatial extent was selected as the scale of effect. Vertical dash bars represent the selected scale of effect (where R^2 is the highest in the original data set).
